# Medical-Legal Partnerships for women in the United States: a systematic review protocol

**DOI:** 10.1186/s13643-025-02956-3

**Published:** 2025-10-24

**Authors:** Sonia Rupcic, Elizabeth Finer, Guneet K. Jasuja, Deborah Gurewich, Melissa E. Dichter, Sonya Borrero

**Affiliations:** 1 Center for Healthcare Evaluation, Research and Promotion, VA Pittsburgh Healthcare System, Pittsburgh, PA USA; 2 Department of Psychology, Hofstra University, Long Island, NY USA; 3 Center for Health Optimization and Implementation Research, VA Bedford Healthcare System, Bedford, MA USA; 4 Section of General Medicine, Chobanian and Avedisian School of Medicine, Boston University, Boston, MA USA; 5 Department of Health Law, Policy and Management, Boston University School of Public Health, Boston, MA USA; 6 Center for Health Optimization and Implementation Research, VA Boston Healthcare System, Boston, MA USA; 7 Center for Healthcare Evaluation, Research and Promotion, Crescenz Philadelphia VA Medical Center, Philadelphia, PA USA; 8 School of Social Work, Temple University, Philadelphia, PA USA; 9 Department of Medicine, University of Pittsburgh, Pittsburgh, PA USA

**Keywords:** Women, Gender, Civil legal needs, Medical-Legal Partnership, Social determinants of health

## Abstract

**Background:**

MLPs offer a promising approach to addressing needs related to adverse social determinants of health through the provision of legal services in clinical healthcare settings. Evidence to support the MLP model is in early stages. Little is known about the gendered impacts of the MLP model or how they might be tailored for women. The objectives of this systematic review are to (1) compare the impacts of Medical-Legal Partnerships (MLPs) by gender, and (2) identify tailored approaches adopted by MLPs to assist women.

**Methods:**

This protocol conforms to the PRISMA-P (Preferred Reporting Items for Systematic Reviews and Meta-Analyses-Protocol) Statement. MEDLINE, Embase, PsycInfo, Web of Science, Scopus, NBER, LexisUni, HeinOnline, and the National Center for Medical Legal Partnerships web site will be searched for English-language manuscripts published after 1993 (the year attributed to the first MLP). For Objective 1, we will apply the PICO (Population, Intervention, Comparators, Outcomes) Framework to determine eligibility, focusing on peer-reviewed articles that describe MLPs in the U.S. (Intervention) that serve women (Population) and present evidence on MLP’s interpersonal-, institutional-, community-, and policy-level impacts (Outcomes) by gender (Comparators). To evaluate bias in Objective 1 articles, we will use the Risk-of-Bias 2 tool for randomized controlled trials and the Risk of Bias in Non-Randomized Studies of Interventions for non-randomized studies. For Objective 2, we will apply the PICo (Population, phenomena of Interest, Context) framework, including peer-reviewed and gray literature that describes tailored approaches (phenomena of Interest) to providing legal support to women or females (Population) in U.S. clinical settings (Context). We will use the Joanna Briggs Institute Critical Appraisal Checklist for Qualitative Research to assess possible bias for Objective 2 articles. Two reviewers will independently screen titles, abstracts, and full texts, meeting regularly to discuss conflicts and establish consensus. Data from selected publications will be extracted and entered in a matrix. Findings will be presented in narrative and tabular forms.

**Discussion:**

This review will provide invaluable evidence on the impact of MLPs by gender and will provide insights into future research and interventions on MLPs serving populations of women.

**Systematic review registration:**

Open Science Framework: https://osf.io/wd6t5/?view_only=2d6baf14c7b2408293ac31ee5921c8e3

**Supplementary Information:**

The online version contains supplementary material available at 10.1186/s13643-025-02956-3.

## Background

Women confront a unique set of social determinants of poor health (SDOH), those health-impacting “conditions in which people are born, grow, live, work and age” [1]. It has been well established that adverse SDOH arise from “the inequitable distribution of power, money, and resources” [1]. Social roles, discrimination, violence, and inequality are patterned in gendered ways. As a result, the quantity, type, and impact of SDOH differs by gender [2, 3]. Women are more likely to live on lower incomes than men, and single women are more likely to head low-income households with children [4]. These economic forms of hardship are associated with high rates of morbidity and mortality [5]. Further, roughly one in four women experience intimate partner violence (IPV) in their lifetimes [6], the health effects of which are devastating and persistent [7, 8]. Gender inequality profoundly shapes the conditions in which different women live, with significant health impacts [9]. Gender’s influence on health is mediated and moderated by other intersecting forms of systemic discrimination, like classism, racism, homophobia, and transphobia. Adverse SDOH tend to operate synergistically. For example, the health of women who experience violence is worsened by the effects of housing instability and economic hardship [10].

Medical-Legal Partnerships (MLP) involve collaborations between clinicians and legal professionals to address adverse SDOH. The MLP movement is said to have started in its current form in 1993, the year Boston Medical Center began offering medical-legal services to low-income families [11]. The movement’s goal was to improve children’s health and wellness by using the law to ensure educational access (e.g., disability accommodations) or households free of environmental hazards (e.g., lead, asbestos) [11]. Other pediatric settings followed suit [11]. While doctor-lawyer alliances have a long history, what distinguishes the MLP model from civil rights-era predecessors [11] is the MLP’s high level of integration with clinical institutions [12]. In its ideal form, MLPs invite legal professionals into clinical settings to provide patients with legal assistance. MLPs further provide clinicians with training in identifying adverse SDOH that have legal remedies. Hundreds of hospitals have embraced this MLP model as a way of meaningfully adopting a holistic, person-centered approach to health care [13].

Early evidence suggests the great promise of MLPs in addressing adverse SDOH. Observational studies find that patients who receive MLP services have improvements in housing stability, access to financial resources, and mental health [14–17]. Much of this research has taken place in pediatric settings, where MLPs got their start [13]. Three trials have used randomization to test the efficacy of MLPs. Two reported on MLPs serving pediatric populations and found increased use of preventative services, decreases in emergency care utilization, and improved diabetes control [16, 18]. A third recruited low-income adults from an urban primary care clinic to be referred to a MLP and found mixed results [19]. While measures of stress and emergency department utilization showed improvement, outcomes in anxiety and hospitalization worsened [19]. In interpreting their findings, investigators noted that medical-legal integration may have been too shallow [19, 20]. However, an alternative interpretation might be that the efficacy of MLPs is limited to certain populations.

Most research on the effects of MLPs on populations of women comes from case studies describing MLPs based in reproductive health clinics that serve cisgender women who are pregnant or postpartum. This literature demonstrates the important role MLPs play in enhancing access to safe and stable housing, reproductive healthcare coverage, public benefits, and restraining orders against abusive partners [21–24]. Little is known about the effects of these services on women beyond individual reproductive care settings. A search of Systematic Reviews, Cochrane Library, and JBI Evidence Synthesis produced no reviews related to MLPs and this patient population. Given the gendered impacts of adverse SDOH, this is a critical gap.

The social identity of woman includes great heterogeneity (e.g., cis versus transgender women, racialization, citizenship status, socioeconomic status). Nevertheless, many MLPs with a defined patient population specialize in serving women [25]. This focus is justified on the grounds that this population confronts unique adverse SDOH and has different experiences accessing aid from the legal sector [26, 27]. Indeed, a recent study suggests that women Veterans have significantly higher legal needs than Veteran men [28]. Women’s legal problems may arise from medical conditions that disproportionately affect women (e.g., abortion access) [29]. Or they may arise from gendered adverse SDOH (e.g., intimate partner violence, needs related to single parenting) [2, 3]. Intersecting adverse SDOH may in turn affect the legal mechanisms more commonly needed by women. For example, compared to men, women are more likely to be single-parenting in poverty, a condition that puts them at heightened risk of having their children taken into foster care on grounds of neglect [30] and means they may have greater need for support in petitioning Child Protective Services or family court [24, 31]. Understanding the outcomes of MLPs on this population and how implementation of this intervention could be tailored to this population has the potential to improve women’s health and wellness by addressing adverse SDOH. This study seeks to review and report on MLPs providing services to women to answer these questions using the Population, Intervention, Comparators, and Outcomes (PICO) [32] and Population, phenomena of Interest, and the Context (PICo) [33] frameworks.

## Methods

### Protocol and registration

This protocol conforms to the 2015 protocol reporting guidelines developed by Moher et al., the Preferred Reporting Items for Systematic Review and Meta-Analysis Protocols (PRISMA-P) Statement (see Additional File 1), a set of guidelines developed to improve the transparency, accuracy, and completeness of systematic reviews and meta-analyses. The protocol is registered with the Open Science Framework at https://osf.io/wd6t5/?view_only=2d6baf14c7b2408293ac31ee5921c8e3.

### Objectives

To enhance understanding of the effects of MLPs on women, this systematic review has two objectives: (1) compare the impacts of Medical-Legal Partnerships (MLPs) by gender, and (2) identify tailored approaches adopted by MLPs to assist women. For Objective 1, we will use the Population, Intervention, Comparators, and Outcomes (PICO) framework to define eligibility criteria as it is well suited to quantitative questions [32]. For Objective 2, we will apply the Population, phenomena of Interest, and the Context (PICo) framework, which is adapted to qualitative questions [33]. It is common to apply both frameworks in a systematic review with both quantitative and qualitative objectives [32, 33].

### Eligibility criteria

#### Population

The systematic review will include publications that describe MLPs that serve women in the United States. We take our cues from the MLPs on how they define this population (e.g., inclusion of transgender as well as cisgender women); we will note how MLPs define their service population. The review will exclude articles that (1) only report on men, or (2) do not mention gender, or (3) are based outside of the U.S.

#### Intervention

MLPs are defined by the presence of eight core elements (see Table 1) [34], but based on our preliminary searches, such criteria are rarely reported upon exhaustively in MLP literature. It is unclear the extent to which most MLPs meet these criteria in practice [12]. We understand MLPs to entail the institutionalized participation of legal professionals on holistic health care teams [11]. We will exclude articles that describe medical-legal collaborations in which legal services are not delivered in the partnered clinical setting. These services need not be continuously co-located in the clinical setting for the article to be included.

**Table 1:** Central components of a Medical-Legal Partnership [34]

1. A formal agreement between the clinical setting and legal services organization that outlines goals, responsibilities and protections for patient privacy and confidentiality
2. A defined patient population (e.g., medical condition and socioeconomic status)
3. A strategy to screen patients for legal needs
4. Dedicated legal professionals to staff the MLP
5. A “lawyer in residence” within the clinical setting or in close proximity to providers
6. Lawyers provide training to providers about identifying health-harming legal needs
7. Screening and referral processes, including information-sharing arrangements
8. Designated budget and resources to support MLP operations

#### Objective 1 (comparators and outcomes)

Objective 1 involves assessing the effectiveness of MLPs by gender. We make use of the traditional Population, Intervention, Comparators, and Outcomes (PICO) approach to frame our eligibility criteria. Population and Intervention are described above. We will include peer-reviewed publications that compare the impact of MLPs by gender and include women as one of the *comparators*. Following proponents of MLPs who stress their important role in addressing structural problems [35], we understand *outcomes* broadly to be measured at the individual, relationship, institution, community, and policy levels. As part of this review, we will consider articles that describe outcomes that affect women across these social-ecological dimensions. For example, individual-level outcomes may include changes to clinically assessed or self-reported health outcomes. Institution-level outcomes may include increased rates of screening for civil legal needs in clinical settings. Policy-level outcomes may include legislative changes. In the case of outcomes on socio-ecological dimensions that extend beyond the individual patient, the publication must make the case that the changes affect health and well-being. To ensure the findings presented on outcomes are as rigorous as possible, the systematic review will only consider peer-reviewed literature for Objective 1, including quantitative studies or mixed method studies with a quantitative aim, that meet the inclusion criteria described above.

#### Objective 2 (phenomenon of interest and context)

Objective 2 involves a qualitative assessment of MLPs tailored to women. We make use of the Population, phenomena of Interest, and the Context (PICo) approach, a framework for conducting systematic reviews adapted to qualitative inquiries [33]. Population is described above. The *phenomenon of interest* guiding Objective 2 is tailoring. It is common for MLPs to develop novel approaches to specific patient populations (e.g., children, homeless patients) [12, 25]. To understand how MLPs tailor services to support women in addressing adverse SDOH, the review will include literature that describes MLP approaches tailored to women. We will include manuscripts that describe tailoring in the *context* of a clinical setting that hosts a MLP. We will include articles that describe MLPs serving all genders, so long as they describe a tailored approach to women. Literature that propounds the value of MLPs for women’s health in abstract terms, without reference to a particular intervention, will be excluded. For Objective 2, this systematic review will include peer-reviewed and gray literature. The latter category might include practice reports that describe the context of one or more “case studies” of a woman-specific MLP describing multiple such approaches. We will consider qualitative, quantitative, or mixed-method studies that meet these criteria. Such literature need not include comparators or report on outcomes.

### Information sources

Because MLPs involve collaborations between clinicians and legal professionals, we will make use of a disciplinary variety of databases, including MEDLINE, Embase, PsycInfo, Web of Science, Scopus, National Bureau of Economic Research, LexisUni, and HeinOnline. Additional studies will be identified from the bibliographies of articles selected for final inclusion and from literature published on the web site of the National Center for Medical Legal Partnerships (NCMLP). We will also connect with legal experts in the MLP field by publicizing this review through the biweekly newsletter operated by the NCMLP and inviting authors and researchers to reach out to the review team with relevant literature. Sources will be searched for English-language manuscripts published after 1993 (the year attributed to the first MLP) [11].

### Search strategy

Our preliminary search strategy was developed iteratively for use in MEDLINE with the assistance of an academic librarian and coauthors with expertise in social needs and women’s health. Search terms for our population were informed by prior systematic reviews on women (see Table 2) [36]. Index articles[16–19] reporting on the health impacts of MLPs were used to identify an initial list of search terms for the intervention, with additional pilot searches generating new keywords, index terms, and Medical Subjects Headings (MeSH) terms (see Table 2).

**Table 2:** Search terms

Construct	Search Term
Medical-Legal Partnership	“medical-legal partnership*” OR “MLP” OR "medical-legal need*” OR "medical-legal service*” OR “medical-legal intervention*” OR “medico-legal service*” OR “medico-legal intervention*” "medical-legal advocacy" OR "health-justice partnership” OR “health justice advocacy” OR “medical-legal integration” OR “legal service” OR “legal advice” OR “civil rights” OR “socio-legal”
AND	
Women’s Health	(MH "Women + ") OR (MH "Battered Women") OR (MH "Single Women") OR (MH "Trans Women") OR (MH "Expectant Mothers") OR (MH "Intimate Partner Violence") OR (MH "Women's Health") OR (MH "Women's Health Services") OR (MH "Lesbians") OR (MH "Breast + ") OR (MH "Breast Neoplasms + ") OR (MH "Breast Self-Examination") OR (MH "Breast Pumps") OR (MH "Breast Tissue Density") OR (MH "Breast Reconstruction") OR (MH "Breast Examination + ") OR (MH "Breast Implants") OR (MH "Breast Feeding + ") OR (MH "Mammography") OR (MH "Mastectomy + ") OR (MH "Genitalia, Female + ") OR (MH "Genital Neoplasms, Female + ") OR (MH "Genital Diseases, Female + ") OR (MH "Female Urogenital Diseases and Pregnancy Complications + ") OR (MH "Sexual Dysfunction, Female + ") OR (MH "Maternal Health Services + ") OR (MH "Prenatal Care") OR (MH "Prenatal Diagnosis + ") OR (MH "Perinatal Period") OR (MH "Perinatal Care") OR (MH "Postnatal Care + ") OR (MH "Postnatal Period + ") OR (MH "Depression, Postpartum") OR (MH "Postpartum Psychosis") OR (MH "Pregnancy + ") OR (MH "Pregnancy Outcomes") OR (MH "Pregnancy Discomforts") OR (MH "Lactation") OR (MH "Lactation Disorders + ") OR (MH "Contraception + ") OR (MH "Hormonal Contraception") OR (MH "Contraceptive Agents + ") OR (MH "Prepregnancy Care") OR (MH "Family Planning + ") OR (MH “Fertility + ") OR (MH "Fertility Preservation") OR (MH "Infertility + ") OR (MH "Obstetric Emergencies") OR (MH "Delivery, Obstetric + ") OR (MH "Obstetric Patients") OR (MH "Obstetric Service") OR (MH "Obstetric Care + ") OR (MH "Surgery, Obstetrical + ") OR (MH "Gynecologic Examination") OR (MH "Surgery, Gynecologic + ") OR (MH "Diagnosis, Gynecologic + ") OR (MH "Gynecologic Care") OR (MH "Abortion, Incomplete") OR (MH "Abortion, Induced + ") OR (MH "Abortion, Spontaneous + ") OR (MH "Menstrual Cycle + ") OR (MH Dysmenorrhea") OR (MH "Menstrual and Perimenopausal Disorders + ") OR (MH "Menstruation Disorders + ") OR (MH "Menstruation") OR (MH "Menstruation Inducing Agents + ") OR (MH "Oligomenorrhea") OR (MH "Menopause + ") OR (MH "Menarche") OR (MH "Menopause, Premature") OR (MH "Premenopause") OR (MH "Postmenopause") OR (MH "Postmenopausal Disorders") OR (MH "Perimenopause") OR (MH "Domestic Violence + ") OR (MH "Rape") OR ((TI woman OR AB woman) OR (TI women OR AB women) OR (TI womens OR AB womens) OR (TI womans OR AB womans) OR (TI "women s" OR AB "womens") OR (TI "woman s" OR AB "woman s") OR (TI female OR AB female) OR (TI females OR AB females) OR (TI "female s" OR AB "female s") OR (TI trans OR AB trans) OR (TI transgender OR AB transgender) OR (TI transgendered OR AB transgendered) OR (TI transfemale OR AB transfemale) OR (TI transfemale OR AB trans-female) OR (TI transman OR AB transman) OR (TI transman OR AB trans-man) OR (TI transmans OR AB transmans) OR (TI"transman s" OR AB "transman s") OR (TI trans-mans OR AB trans-mans) OR (TI "trans-man s" OR AB "trans-man s") OR (TI transmen OR AB transmen) OR (TI trans-men OR AB trans-men) OR (TI transmens OR AB transmens) OR (TI trans-mens OR AB trans-mens) OR (TI "transmen s" OR AB "transmen s") OR (TI "transmen s" OR AB "transmen s") OR (TI transwoman OR AB transwoman) OR (TI trans-woman OR AB trans-woman) OR (TI transwomans OR AB transwomans) OR (TI "transwoman s" OR AB "transwoman s") OR (TI trans-womans OR AB trans-womans) OR (TI "trans-woman s" OR AB "transwoman s") OR (TI trans-women OR AB trans-women) OR (TI transwomen OR AB transwomen) OR (TI transwomens OR AB transwomens) OR (TI "transwomen s" OR AB "transwomen s") OR (TI trans-womens OR AB transwomens) OR (TI "transwomen s" OR AB "transwomen s") OR (TI genderspecific OR AB gender-specific) OR (TI "gender specific" OR AB "gender specific") OR (TI gender-related OR AB gender-related) OR (TI "gender related" OR AB "gender related") OR (TI "gender difference" OR AB "gender difference") OR (TI "gender differences" OR AB "gender differences") OR (TI sex-specific OR AB sex-specific) OR (TI "sex specific" OR AB "sex specific") OR (TI sex-related OR AB sex-related) OR (TI "sex related" OR AB "sex related") OR (TI "sex difference" OR AB "sex difference") OR (TI "sex differences" OR AB "sex differences") OR (TI lesbian OR AB lesbian) OR (TI lesbians OR AB lesbians) OR (TI non-binary OR AB non-binary) OR (TI "non binary" OR AB "non binary") OR (TI abortifacient OR AB abortifacient) OR (TI abortifacients OR AB abortifacients) OR (TI abortion OR AB abortion) OR (TI abortions OR AB abortions) OR (TI amenorrhea OR AB amenorrhea) OR (TI breast OR AB breast) OR (TI breasts OR AB breasts) OR (TI breastfeeding OR AB breastfeeding) OR (TI cervix OR AB cervix) OR (TI cervical OR AB cervical) OR (TI climacteric OR AB climacteric) OR (TI clitoris OR AB clitoris) OR (TI clitoral OR AB clitoral) OR (TI colposcop* OR AB colposcop*) OR (TI colpotom* OR AB colpotom*) OR (TI conception OR AB conception) OR (TI contraception OR AB contraception) OR (TI contraceptive OR AB contraceptive) OR (TI contraceptives OR AB contraceptives) OR (TI culdoscop* OR AB culdoscop*) OR (TI dysmenorrhea OR AB dysmenorrhea) OR (TI dyspareunia OR AB dyspareunia) OR (TI endometriosis OR AB endometriosis) OR (TI endometritis OR AB endometritis) OR (TI endometrium OR AB endometrium) OR (TI endometrial OR AB endometrial) OR (TI endometrioid OR AB endometrioid) OR (TI episiotom* OR AB episiotom*) OR (TI fallopian OR AB fallopian) OR (TI fallopians OR AB fallopians) OR (TI "family planning" OR AB "family planning") OR (TI fertility OR AB fertility) OR (TI gynecolog* OR AB gynecolog*) OR (TI "hot flash" OR AB "hot flash") OR (TI "hot flashes" OR AB "hot flashes") OR (TI hymen OR AB hymen) OR (TI hymens OR AB hymens) OR (TI hysterectom* OR AB hysterectom*) OR (TI hysteroscop* OR AB hysteroscop*) OR (TI infertility OR AB infertility) OR (TI "intimate partner violence" OR AB "intimate partner violence") OR (TI "intrauterine device" OR AB "intrauterine device") OR (TI "intrauterine devices" OR AB "intrauterine devices") OR (TI IUD OR AB IUD) OR (TI IUDs OR AB IUDs) OR (TI labia OR AB labia) OR (TI labias OR AB labias) OR (TI labial OR AB labial) OR (TI lactation OR AB lactation) OR (TI lactating OR AB lactating) OR (TI mammaplast* OR AB mammaplast*) OR (TI mammoplast* OR AB mammoplast*) OR (TI mammogra* OR AB mammogra*) OR (TI mastectom* OR AB mastectom*) OR (TI maternal OR AB maternal) OR (TI maternally OR AB maternally) OR (TI menopaus* OR AB menopaus*) OR (TI menorrhagia OR AB menorrhagia) OR (TI menstrua* OR AB menstrua*) OR (TI menses OR AB menses) OR (TI menarche OR AB menarche) OR (TI "military sexual trauma" OR AB "military sexual trauma") OR (TI "military sexual assault" OR AB "military sexual assault") OR (TI "morning after pill" OR AB "morning after pill") OR (TI "morning after pills" OR AB "morning after pills") OR (TI obstetric* OR AB obstetric*) OR (TI oligomenorrhea OR AB oligomenorrhea) OR (TI oophorectom* OR AB oophorectom*) OR (TIoophoritis OR AB oophoritis) OR (TI ovariectom* OR AB ovariectom*) OR (TI ovary OR AB ovary) OR (TI ovaries OR AB ovaries) OR (TI ovarian OR AB ovarian) OR (TI "painful period" OR AB "painful period") OR (TI "painful periods" OR AB "painful periods") OR (TI "irregular period" OR AB "irregular period") OR (TI "irregular periods" OR AB "irregular periods") OR (TI PCOS OR AB PCOS) OR (TI perimenopaus* OR AB perimenopaus*) OR (TI perimenopaus* OR AB peri-menopaus*) OR (TI perinatal OR AB perinatal) OR (TI peri-natal OR AB peri-natal) OR (TI perinatally OR AB perinatally) OR (TI perinatally OR AB peri-natally) OR (TI PMDD OR AB PMDD) OR (TI postmenopaus* OR AB postmenopaus*) OR (TI post-menopaus* OR AB postmenopaus*) OR (TI postnatal OR AB postnatal) OR (TI post-natal OR AB postnatal) OR (TI postnatally OR AB postnatally) OR (TI post-natally OR AB postnatally) OR (TI postpartum OR AB postpartum) OR (TI post-partum OR AB post-partum) OR (TI preconception OR AB preconception) OR (TI preconception OR AB pre-conception) OR (TI pregnancy OR AB pregnancy) OR (TI pregnancies OR AB pregnancies) OR (TI pregnant OR AB pregnant) OR (TI pregnancy-induced OR AB pregnancy-induced) OR (TI pregnancy-associated OR AB pregnancy-associated) OR (TI prepregnancy OR AB prepregnancy) OR (TI premenstrual OR AB premenstrual) OR (TI pre-menstrual OR AB premenstrual) OR (TI prenatal OR AB prenatal) OR (TI pre-natal OR AB pre-natal) OR (TI prenatally OR AB prenatally) OR (TI pre-natally OR AB pre-natally) OR (TI puerperium OR AB puerperium) OR (TI rape OR AB rape) OR (TI rapes OR AB rapes) OR (TI raped OR AB raped) OR (TI "reproductive health" OR AB "reproductive health") OR (TI "reproductive care" OR AB "reproductive care") OR (TI "reproductive healthcare" OR AB "reproductive healthcare") OR (TI "reproductive plan" OR AB "reproductive plan") OR (TI "reproductive planning" OR AB “reproductive planning") OR (TI salpingectom* OR AB salpingectom*) OR (TI salpingo-oophorectom* OR AB salpingo-oophorectom*) OR (TI uterus OR AB uterus) OR (TI uterine OR AB uterine) OR (TI vagina OR AB vagina) OR (TI vaginas OR AB vaginas) OR (TI vaginal* OR AB vaginal*) OR (TI transvaginal* OR AB transvaginal*) OR (TI vaginismus OR AB vaginismus) OR (TI vulva OR AB vulva) OR (TI vulvas OR AB vulvas) OR (TI vulvar OR AB vulvar) OR (TI vulvectom* OR AB vulvectom*) OR (TI vulvitis OR AB vulvitis) OR (TI vulvodynia OR AB vulvodynia)) OR (((TI dilatation OR AB dilatation) OR (TI vacuum OR AB vacuum)) N2 (TI curettage OR AB curettage)) OR (((TI sex OR AB sex) OR (TI sexual OR AB sexual) OR (TI sexually OR AB sexually) OR (TI domestic OR AB domestic) OR (TI partner OR AB partner) OR (TI spouse OR AB spouse) OR (TI spousal OR AB spousal) OR (TI physical OR AB physical) OR (TI physically OR AB physically)) N3 ((TI abuse OR AB abuse) OR (TI abuses OR AB abuses) OR (TI abused OR AB abused) OR (TI abuser OR AB abuser) OR (TI abusers OR AB abusers) OR (TI abusive OR AB abusive) OR (TI violence OR AB violence) OR (TI violent OR AB violent) OR (TI assault OR AB assault) OR (TI assaults OR AB assaults) OR (TI assaulted OR AB assaulted))) OR ((TI tubal OR AB tubal) N2 ((TI ligation* OR AB ligation*) OR (TI sterilization* OR AB sterilization*) OR (TI sterilisation* OR AB sterilisation*)))

### Study records

#### Data management

We will initially assemble all articles into EndNote v.21 reference software before transferring them to Covidence, an online review management software designed to facilitate collaborative literature reviews. We will select, review, and code literature using Covidence. The selection process will be documented in Covidence. Data from publications selected in Covidence will be extracted and entered in an Excel matrix. Findings will be presented in narrative and tabular forms.

#### Selection process

In Covidence, SR and EF will remove duplicates and engage in study selection. We will review titles and abstracts (or full texts, in the case of articles without available abstracts) and exclude articles based on the aforementioned criteria. We will pilot test the first 10 articles to ensure the inclusion and exclusion criteria are being applied consistently. For the pilot testing, SR and EF will independently screen 10 articles and then meet to ensure consistent application of inclusion/exclusion criteria, develop rules for applying criteria, and identify inductive themes. Unresolved conflicts will be presented to the larger study team. Thereafter, SR and EF will continue to screen titles and abstracts independently. After reviewing all titles and abstracts, SR and EF will screen the full text of potentially relevant articles against all inclusion and exclusion criteria. Included studies will be tagged for relevance for Objective 1, Objective 2, or both. The bibliographies of publications included for full text review will be screened and tagged in Covidence to identify additional articles for further screening. We will meet regularly to discuss any conflicts identified in Covidence, consulting a third team member on any decisions where we cannot achieve consensus.

#### Data collection process

Data extraction will involve a process of deductive and inductive analysis. First, we will develop a data extraction template in Covidence to deductively extract the data items defined below. SR and EF will review each article independently. After completing data extraction for the first 20% of articles, we will meet to verify deductive categories are being applied consistently, discuss differences in coding, and establish rules to ensure consistency. We will also consider additional data items that emerge inductively as part of the pilot review to incorporate in subsequent reviews and to guide additional data collection from previously reviewed articles. We will repeat this process for the next 20% of articles and again for the remaining articles until we have completed data collection. If necessary, a third team member will resolve any discrepancies in coding.

### Data items

For each article, we will collect high-level Study Characteristics, including author(s), publication date, state(s) where MLP operates, clinical setting of MLP, a description of the MLP’s target patient population, whether the eight core elements of a MLP are satisfied [12], whether the article evaluates outcomes by gender (Objective 1), describes one or more case studies of MLPs tailored to women (Objective 2), or does both (Objectives 1 and 2). For each article, we will extract data to make determinations about risks of biases. For publications tagged “Objective 1,” or “Objectives 1 and 2,” we will extract data on the study design, total sample size, genders compared with sub-sample sizes, type of outcomes, and effect size. For publications tagged “Objective 2” or “Objectives 1 and 2,” we will also describe how the intervention is tailored to women. As data collection progresses, we will develop new codes to represent inductively identified data items.

### Outcomes and prioritization

MLPs affect health indirectly by addressing adverse SDOH. For that reason, we are interested in a broad array of outcomes. No one outcome will be given priority in data analysis and synthesis.

### Risk of bias in individual studies

Because different data for Objectives 1 and 2 may be extracted from a single publication, multiple types of assessments may be conducted for a single publication.

#### Objective 1

We will make use of different risk of bias assessment tools for Objective 1. Randomized controlled trials (RCTs) will be assessed with the Risk-of-Bias 2 (RoB-2) tool [37, 38], developed for use in systematic reviews to evaluate biases in RCTs across a variety of domains. Because the RoB-2 is used for individual RCT outcomes, several RoB-2 assessments may need to be completed for each RCT. For non-randomized studies, we will use the Risk of Bias in Non-Randomized Studies of Interventions (ROBINS-I) tool, which evaluates biases in studies that report estimates of comparative effectiveness but do not use randomization. Judgements about bias will be accompanied by written justifications.

#### Objective 2

For the review of qualitative data about MLP tailoring, we will make use of the Joanna Briggs Institute (JBI) Critical Appraisal Checklist for Qualitative Research [33]. This JBI checklist of ten items captures multiple domains of validity and has the added benefit of focusing on whether a given study demonstrates congruity between its stated philosophical perspective and its research methodology, a critical feature given the diversity of epistemological assumptions undergirding different qualitative research approaches [39]. Checklist items will be accompanied by written justification.

### Data synthesis

Preliminary searches suggest that existing literature varies widely in the types of outcomes reported [40].As such, we do not plan to conduct meta-analyses. Data synthesis will be qualitative and narrative. We will export extracted data from Covidence into an Excel spreadsheet. We will conduct two qualitative matrix analyses to identify themes in deductive and inductive data items pertaining to outcomes (Objective 1) and MLP tailoring (Objective 2). For the outcomes analysis (Objective 1), special attention will be paid to the relationship between outcome and tailoring, if relevant. Drawing on our qualitative analyses, we will summarize our findings on the gendered effects of MLPs and how MLPs tailor their services to women, identifying gaps in the literature and implications for future research. We will report the results of the data screening and review using PRISMA guidelines.

### Confidence in cumulative evidence

For Objective 1, we will evaluate the strength of the body of evidence for each outcome using the Grading of Recommendations Assessment, Development, and Evaluation (GRADE) system of rating the quality of evidence [41]. The GRADE system delineates five criteria that can reduce the quality rating: study limitations, imprecision, inconsistency of results, indirectness of evidence, and likely publication bias. GRADE further delineates three criteria that can increase the rating of the quality of a given study’s evidence: large magnitude of effect, dose response, and confounders that likely minimize the effect. Research on MLPs is limited. To date, only three RCTs on MLPs have been completed [19]. Because the GRADE system weights RCTs as high-quality study designs, we do not anticipate identifying multiple high-quality studies. Following the GRADE scale, we will rank the studies as high, moderate, low, or very low. The review will include a table documenting the factors that went into our assessment of each study.

### Timeline

We plan to complete this review within 1 year. The search will take 1–2 months. Data extraction and synthesis will take 6–8 months. We will complete data summarization and presentation in 1–2 months.

## Discussion

In randomized controlled trials, MLPs have had positive effects on health and healthcare utilization in pediatric populations [16, 18], but mixed results in low-income adults [19]. Taken together, these findings raise questions about the efficacy of this intervention across populations and of different implementation strategies tailored to distinct populations [20]. Women confront a host of unique needs arising from adverse SDOH. And yet, little is known about how best to implement Medical-Legal Partnerships for this group. The proposed systemic review will provide invaluable evidence about whether and how best to implement MLPs for women.

## Supplementary Information

Additional file 1.

## Data Availability

Data sharing is not applicable to this article as no datasets were generated or analyzed for this protocol.

## Abbreviations

GRADE: Grading of Recommendations Assessment, Development, and Evaluation
JBI: Joanna Briggs Institute
MLP: Medical-Legal Partnerships
NCMLP: National Center for Medical Legal Partnerships
PICO: Population, Intervention, Comparators, and Outcomes
PICo: Population, phenomena of Interest, and the Context
PRISMA: Preferred Reporting Items for Systematic Review and Meta-Analysis Protocols
RCT: Randomized controlled trials
RoB-2: Risk-of-Bias 2
ROBINS-I: Risk of Bias in Non-Randomized Studies of Interventions
SDOH: Social Determinants of Health

## Declarations

Ethics approval and consent to participate

Not applicable.

## References

[1] Commission on Social Determinants of Health. Closing the gap in a generation: health equity through action on the social determinants of health. Geneva: World Health Organization; 2008 p. 247. Available from: https://iris.who.int/handle/10665/43943. Cited 2024 May 6. 10.1016/S0140-6736(08)61690-6 18994664

[2] Ballantyne PJ. The social determinants of health: a contribution to the analysis of gender differences in health and illness. Scand J Public Health. 1999;27(4):290–5. 10724473

[3] Byhoff E, Tripodis Y, Freund KM. Gender differences in social and behavioral determinants of health in aging adults. J Gen Intern Med. 2019;34(11):2310–2. 31385211 10.1007/s11606-019-05225-x PMC6848471

[4] U.S. Census Bureau. Current Population Survey, 2023, Annual Social and Economic Supplement. Washington, D.C: U.S. Census Bureau; 2023. Available from: https://www.census.gov/data/datasets/time-series/demo/cps/cps-asec.html.

[5] Chokshi DA. Income, poverty, and health inequality. JAMA. 2018;319(13):1312–3. 29614168 10.1001/jama.2018.2521

[6] Black MC. Intimate partner violence and adverse health consequences: implications for clinicians. Am J Lifestyle Med. 2011;5(5):428–39.

[7] National Center for Injury Prevention and Control. Costs of Intimate Partner Violence Against Women in the United States. Atlanta, GA: Centers for Disease Control and Prevention; 2003. Available from: https://www-cdc-gov.revproxy.brown.edu/violenceprevention/pub/IPV_cost.html. Cited 2024 May 3.

[8] Iverson KM, Pogoda TK. Traumatic brain injury among women veterans: an invisible wound of intimate partner violence. Med Care. 2015;53(4 Suppl 1):S112-119. 25767964 10.1097/MLR.0000000000000263

[9] Cannon Y. Closing the health justice gap: access to justice in furtherance of health equity. Columbia Hum Rights Law Rev. 2021;2022(53):517.

[10] Daoud N, Matheson FI, Pedersen C, Hamilton-Wright S, Minh A, Zhang J, et al. Pathways and trajectories linking housing instability and poor health among low-income women experiencing intimate partner violence (IPV): Toward a conceptual framework. Women Health. 2016 Feb 17; Available from: 10.1080/03630242.2015.1086465. Cited 2024 Apr 24. 26358378

[11] Teitelbaum J, Lawton E. The roots and branches of the medical-legal partnership approach to health: from collegiality to civil rights to health equity. Yale J Health Policy Law Ethics. 2017;17(2):343.

[12] Regenstein M, Trott J, Williamson A, Theiss J. Addressing social determinants of health through medical-legal partnerships. Health Aff. 2018;37(3):378–85. 10.1377/hlthaff.2017.1264 29505366

[13] Matthew DB, Benfer EA. A clarion call for change: the MLP imperative to center racial discrimination and structural health inequities. J Law Med Ethics. 2023;51(4):735–47. 38477284 10.1017/jme.2023.153 PMC10951182

[14] Rosen Valverde JN, Backstrand J, Hills L, Tanuos H. Medical-legal partnership impact on parents’ perceived stress: a pilot study. Behav Med. 2019;45(1):70–7. 29944063 10.1080/08964289.2018.1481011

[15] Ryan AM, Kutob RM, Suther E, Hansen M, Sandel M. Pilot study of impact of medical-legal partnership services on patients’ perceived stress and wellbeing. J Health Care Poor Underserved. 2012;23(4):1536–46. 23698668 10.1353/hpu.2012.0179

[16] Malik FS, Yi-Frazier JP, Taplin CE, Roth CL, Whitlock KB, Howard W, et al. Improving the care of youth with type 1 diabetes with a novel medical-legal community intervention: the diabetes community care ambassador program. Diabetes Educ. 2018;44(2):168–77. 29320934 10.1177/0145721717750346

[17] Tsai J, Middleton M, Villegas J, Johnson C, Retkin R, Seidman A, et al. Medical-legal partnerships at Veterans Affairs medical centers improved housing and psychosocial outcomes for vets. Health Aff Millwood. 2017;36(12):2195–203. 29200329 10.1377/hlthaff.2017.0759

[18] Sege R, Preer G, Morton SJ, Cabral H, Morakinyo O, Lee V, et al. Medical-legal strategies to improve infant health care: a randomized trial. Pediatrics. 2015;136(1):97–106. 26034248 10.1542/peds.2014-2955 PMC9923600

[19] Liaw W, Northrup TF, Stotts AL, Bakos-Block C, Suchting R, Chen A, et al. Medical-legal partnership effects on mental health, health care use, and quality of life in primary care: a randomized clinical trial. J Am Board Fam Med. 2023;36(3):414–24. 37028914 10.3122/jabfm.2022.220349R1

[20] Liaw W, Bakos-Block C, Northrup TF, Stotts AL, Hernandez A, Finzetto L, et al. A Qualitative Analysis of a Primary Care Medical-Legal Partnership: Impact, Barriers, and Facilitators. J Am Board Fam Med. 2024 Aug 30; Available from: https://www.jabfm.org/content/early/2024/08/30/jabfm.2023.230328R2. Cited 2024 Oct 21. 10.3122/jabfm.2023.230328R2 39214697

[21] Patchen L, Richardson R, McCullers A, Girard V. Integrating lawyers into perinatal care teams to address unmet, health-harming legal needs. Obstet Gynecol. 2023;142(6):1310. 37884009 10.1097/AOG.0000000000005417 PMC10642697

[22] Jones G, Goulland L. The last piece of the puzzle that makes all the difference in the world:” team-facing medical-legal partnership for reproductive care teams. J Law Med Ethics. 2023;51(4):865–73. 38477280 10.1017/jme.2024.16

[23] Gievers L, Mutrie L, Klawetter S. Novel perinatal medical-legal partnership development and pilot implementation to address health-harming legal needs. J Perinatol. 2024;44(1):136–41. 38007591 10.1038/s41372-023-01829-8 PMC10872264

[24] Finck KR, Greenberg S. The family regulation system and medical-legal partnerships. J Law Med Ethics. 2023;51(4):831–7. 38477264 10.1017/jme.2024.2 PMC10937172

[25] Timko C, Taylor E, Nash A, Blonigen D, Smelson D, Tsai J, et al. National survey of legal clinics housed by the department of veterans affairs to inform partnerships with health and community services. J Health Care Poor Underserved. 2020;31(3):1440–56. 33416704 10.1353/hpu.2020.0104 PMC8215811

[26] Selnau K, Goldberg RC. Medical-Legal Partnerships in the VA. In: Tsai J, Seamone ER, editors. Intersections between Mental Health and Law among Veterans [Internet]. Cham: Springer International Publishing; 2019. p. 59–87. Available from: 10.1007/978-3-030-31664-8_4. Cited 2024 Apr 22.

[27] Rubin R. Medical-legal partnerships: how legal services can dramatically improve health outcomes, and the missed opportunity to help women seeking abortions family law writing competition winner. Fam Court Rev. 2019;57(4):569–82.

[28] Gurewich D, Shoushtari SI, Ostrow R, MacLaren RZ, Li M, Harvey K, et al. Prevalence and determinants of unmet social needs among rural and urban veterans. J Health Care Poor Underserved. 2023;34(1):275–92. 37464494 10.1353/hpu.2023.0018

[29] de Londras F, Cleeve A, Rodriguez MI, Farrell A, Furgalska M, Lavelanet AF. The impact of provider restrictions on abortion-related outcomes: a synthesis of legal and health evidence. Reprod Health. 2022;19(1):95. 35436888 10.1186/s12978-022-01405-x PMC9014563

[30] Roberts D. Torn Apart: How the Child Welfare System Destroys Black Families--and How Abolition Can Build a Safer World. Basic Books; 2022. 385 p.

[31] Campbell S. Medical-legal partnerships as tools to reduce child welfare contact: shifting health care providers from sites of surveillance to sites of support notes. Georget J Poverty Law Policy. 2023;31(1):93–120.

[32] McKenzie JE, Brennan SE, Ryan RE, Thomson HJ, Johnston RV, Thomas J. Defining the criteria for including studies and how they will be grouped for the synthesis. In: Higgins JPT, Thomas J, Chandler J, Cumpston M, Li T, Page MJ, et al., editors. Cochrane Handbook for Systematic Reviews of Interventions. 2024. Available from: https://training.cochrane.org/handbook. Cited 2025 Jun 3.

[33] Lockwood C, Munn Z, Porritt K. Qualitative research synthesis: methodological guidance for systematic reviewers utilizing meta-aggregation. Int J Evid Based Healthc. 2015;13(3):179. 26262565 10.1097/XEB.0000000000000062

[34] Tobin-Tyler E, Teitelbaum JB. Medical-legal partnership: a powerful tool for public health and health justice. Public Health Rep. 2019;134(2):201–5. 30644791 10.1177/0033354918824328 PMC6410480

[35] Murphy C. Making the Case for Medical-Legal Partnerships: an updated review of the evidence, 2013–2020. Washington, D.C: National Center for Medical Legal Partnerships; 2020. Available from: https://medical-legalpartnership.org/wp-content/uploads/2020/10/MLP-Literature-Review-2013-2020.pdf.

[36] Pace R, Dancu C, Raman S. An Evidence Map of the Women Veterans’ Health Literature (2016–2023). Washington, DC: Evidence Synthesis Program, Health Systems Research, Office of Research and Development, Department of Veterans Affairs; 2024. Report No.: VA ESP Project #09–01.

[37] Sterne JAC, Savović J, Page MJ, Elbers RG, Blencowe NS, Boutron I, et al. RoB 2: a revised tool for assessing risk of bias in randomised trials. BMJ. 2019;28(366):l4898. 10.1136/bmj.l4898 31462531

[38] Higgins JP, Savović J, Page MJ, Elbers RG, Sterne JA. Chapter 8: Assessing risk of bias in a randomized trial. In: Higgins JP, Thomas J, Chandler J, Cumpston M, Li T, Page M, et al., editors. Cochrane Handbook for Systematic Reviews of Interventions. 2024. Available from: https://training.cochrane.org/handbook/current/chapter-25. Cited 2024 Dec 6.

[39] Hannes K, Lockwood C, Pearson A. A comparative analysis of three online appraisal instruments’ ability to assess validity in qualitative research. Qual Health Res. 2010;20(12):1736–43. 20671302 10.1177/1049732310378656

[40] Martinez O, Boles J, Muñoz-Laboy M, Levine EC, Ayamele C, Eisenberg R, et al. Bridging health disparity gaps through the use of medical legal partnerships in patient care. J Law Med Ethics J Am Soc Law Med Ethics. 2017;45(2):260–73. 10.1177/1073110517720654 PMC7540942 33033428

[41] Guyatt G, Oxman AD, Aki EA, Kunz R, Vist G, Brozek J, et al. GRADE guidelines: 1. Introduction—GRADE evidence profiles and summary of findings tables - ClinicalKey. J Clin Epidemiol. 2011;64(4):383–94. 10.1016/j.jclinepi.2010.04.026 21195583

